# Visualization of automatically combined disease maps and pathway diagrams for rare diseases

**DOI:** 10.3389/fbinf.2023.1101505

**Published:** 2023-07-12

**Authors:** Piotr Gawron, David Hoksza, Janet Piñero, Maria Peña-Chilet, Marina Esteban-Medina, Jose Luis Fernandez-Rueda, Vincenza Colonna, Ewa Smula, Laurent Heirendt, François Ancien, Valentin Groues, Venkata P. Satagopam, Reinhard Schneider, Joaquin Dopazo, Laura I. Furlong, Marek Ostaszewski

**Affiliations:** ^1^ Luxembourg Centre for Systems Biomedicine (LCSB), University of Luxembourg, Luxembourg, Luxembourg; ^2^ Faculty of Mathematics and Physics, Charles University, Prague, Czechia; ^3^ Research Programme on Biomedical Informatics (GRIB), Hospital del Mar Medical Research Institute (IMIM), Barcelona, Spain; ^4^ Department of Experimental and Health Sciences, Pompeu Fabra University (UPF), Barcelona, Spain; ^5^ MedBioinformatics Solutions SL, Barcelona, Spain; ^6^ Computational Medicine Platform, Fundacion Progreso y Salud, Sevilla, Spain; ^7^ Spanish Network of Research in Rare Diseases (CIBERER), Sevilla, Spain; ^8^ Institute of Genetics and Biophysics, National Research Council of Italy, Naples, Rome; ^9^ Department of Genetics, Genomics and Informatics, College of Medicine, University of Tennessee Health Science Center, Memphis, TN, United States

**Keywords:** pathway diagrams, systems biomedicine, rare diseases (RD), disease maps, gene-disease association

## Abstract

**Introduction:** Investigation of molecular mechanisms of human disorders, especially rare diseases, require exploration of various knowledge repositories for building precise hypotheses and complex data interpretation. Recently, increasingly more resources offer diagrammatic representation of such mechanisms, including disease-dedicated schematics in pathway databases and disease maps. However, collection of knowledge across them is challenging, especially for research projects with limited manpower.

**Methods:** In this article we present an automated workflow for construction of maps of molecular mechanisms for rare diseases. The workflow requires a standardized definition of a disease using Orphanet or HPO identifiers to collect relevant genes and variants, and to assemble a functional, visual repository of related mechanisms, including data overlays. The diagrams composing the final map are unified to a common systems biology format from CellDesigner SBML, GPML and SBML+layout+render. The constructed resource contains disease-relevant genes and variants as data overlays for immediate visual exploration, including embedded genetic variant browser and protein structure viewer.

**Results:** We demonstrate the functionality of our workflow on two examples of rare diseases: Kawasaki disease and retinitis pigmentosa. Two maps are constructed based on their corresponding identifiers. Moreover, for the retinitis pigmentosa use-case, we include a list of differentially expressed genes to demonstrate how to tailor the workflow using omics datasets.

**Discussion:** In summary, our work allows for an ad-hoc construction of molecular diagrams combined from different sources, preserving their layout and graphical style, but integrating them into a single resource. This allows to reduce time consuming tasks of prototyping of a molecular disease map, enabling visual exploration, hypothesis building, data visualization and further refinement. The code of the workflow is open and accessible at https://gitlab.lcsb.uni.lu/minerva/automap/.

## 1 Introduction

Investigation of causal mechanisms behind complex diseases is challenging, and one of key components of such investigations is knowledge about implicated molecular mechanisms and pathways. Pathway databases like Reactome or WikiPathways (Martens et al., 2021; Gillespie et al., 2022) offer diagrammatic representations of such mechanisms, also for more prevalent diseases. Moreover, the recent emergence of different disease maps (Singh et al., 2020; Mazein et al., 2021; Ostaszewski et al., 2021) demonstrates the need for disease-oriented repositories of graphical knowledge to support computational pipelines.

At the same time, building disease maps or pathway diagrams requires a substantial curation effort, challenging for small research groups. Especially affected is the area of research on rare diseases (RDs), as these disorders are not prevalent enough to be represented in major bioinformatics resources or pathway databases. However, existing disease-focused resources may offer insights into specific RDs, if searched systematically. Thanks to available tools for translation of standard diagram formats and programmatic interfaces for querying interaction databases it is possible to integrate pieces of multiple data sources into on-the-fly prototype diagrams. For such diagrams to be accurate, it is necessary to precisely formulate search queries, for instance by incorporating standardized phenotypic descriptions of a particular disease, by using genotyping information or relevant omics readouts.

In this article we present a data-driven workflow for on-the-fly building visual and computational repositories of molecular disease mechanisms, focusing in particular on RDs. To this end, we query and combine contents of existing open access repositories of disease-related mechanisms. To ensure precision of the queries we relied on standardized definitions and encoding of disease phenotypes using Orphanet (Orpha, 1997) and Human Phenotype Ontology (Köhler et al., 2021). Users can specify their disease or its phenotypic description based on these resources, which then is used to identify genes and variants relevant for the disease mechanisms. Using these relevant genes, the workflow identifies enriched, publicly available pathway databases and disease maps, together with text mining results, to combine them into a custom disease map, generated on-the-fly. The resulting map is ready to upload on an online visualization platform for further exploration and analysis. Using our workflow, a researcher is able to define an RD of choice, or encode its phenotype, to generate a relevant disease map prototype for further refinement.

## 2 Materials and methods

The workflow for ad-hoc map building is organized into three stages, described in detail below. All indicated parameters below can be set in the workflow configuration files.

1. Disease context: Identify disease-related genes and variants for a given RD
   a. Get gene-and variant-disease mapping from DisGeNET and OpenTargets
    *Configurable parameters*:
      - OrphaNet or HPO identifiers
      - DisGeNET maximum number and score for retrieved genes
      - OpenTargets association score threshold
   b. Get pathogenic variants and genes for the disease from ClinVar
   c. Filter out variants with high allele frequency using Ensemble’s VEP service
*Configurable parameters*:
      - VEP threshold
   d. Compile a summary list of genes and variants associated with the disease
2. Network of mechanisms: Collect disease maps, pathways and networks enriched for disease-related genes
   a. Get enriched diagrams from disease maps
    *Configurable parameters*:
      - Disease map instances (MINERVA Net identifiers)
    - Maximum number of retrieved diagrams
   b. Get enriched diagrams from pathway databases
    *Configurable parameters*:
      - Pathway databases (EnrichR identifiers)
    - Maximum number of retrieved diagrams per database
   a. Construct a text mining network using STRING and OmniPath
    *Configurable parameters*:
      - Maximum number of new neighbors in STRING network
      - Maximum score of retrieved STRING interactions
3. Interactive prototype: Assemble and visualize the prototype map
   a. Compile the obtained pathways into a single diagram
   b. Store gene names for data overlay in the MINERVA Platform
   c. Store variant information (position, protein-level mapping) for genetic variant overlay in the MINERVA Platform
   d. Bundle the disease map with genetic and variant overlays into a single archive to be then uploaded to the MINERVA Platform

**FIGURE 1:**
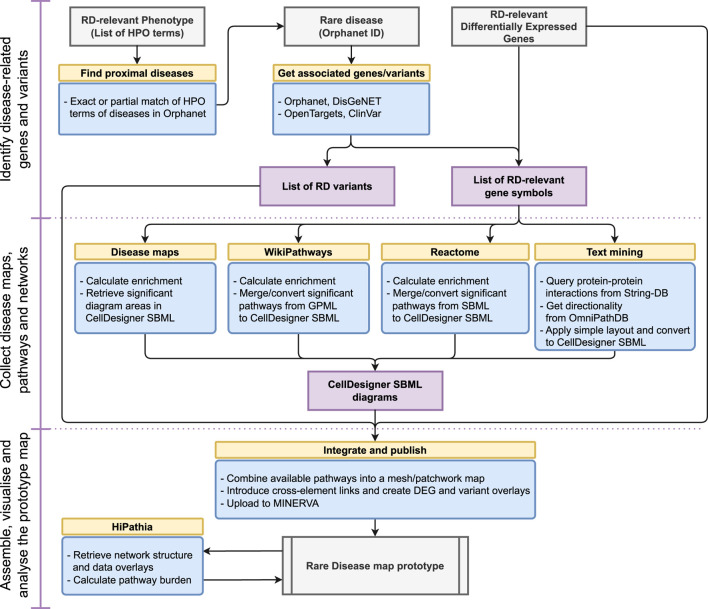
A workflow for ad-hoc map building for rare diseases.

These steps are linked into a single, executable and reproducible workflow, illustrated in Figure 1. Below we discuss resources and methods used in each step. All code, including the integration of particular steps into a workflow, is available in the open repository: https://gitlab.lcsb.uni.lu/minerva/automap/

The repository features a Docker container, facilitating the execution of the workflow. Moreover, we set up a dedicated website under https://automap.elixir-luxembourg.org allowing users to run the workflow using a graphical interface. The number of parameters configurable via the website is limited for the performance reasons, and the users interested in more resource-consuming map builds are encouraged to run the workflow locally using the Docker container.

### 2.1 Disease context

By disease context we understand a list of genes and gene variants associated with a given disease. To obtain such a list, we use two resources: Orphanet and Human Phenotype Ontology (HPO) (Orpha, 1997; Köhler et al., 2021), allowing for unique identification of a disease. The first step of the workflow takes one or more Orphanet identifiers (ORDO) related to a given RD (configurable parameter). For RDs that have no Orphanet identifier, the workflow takes as an input a list of HPO terms describing disease symptoms, e.g., HP:0001644,HP:0002617 for “dilated cardiomyopathy” and “vascular dilatation” (configurable parameter). These identifiers are then matched against the Orphanet database of phenotype-disease associations (http://www.orphadata.org) to find a subset of the most matching Orphanet identifiers.

Having a given set of Orphanet identifiers, we obtain the list of relevant genes and variants by combining:

i) gene-disease mapping of Orphanet
ii) gene-disease and variant-disease mapping from DisGeNET (Piñero et al., 2020)
iii) variant-disease mapping of OpenTargets platform (Carvalho-Silva et al., 2019)
iv) variant-disease mapping of ClinVar (Landrum et al., 2018).

Moreover, disease-associated variants can be filtered for rarity using population allele frequencies obtained from Ensembl Variant Effect Predictor (VEP) (McLaren et al., 2016).

All queries are automated to use respective API endpoints for a pulldown of gene and variant lists. These two lists are considered as the context of the molecular mechanisms of a given disease. To obtain the disease-associated genes and variants, the workflow implements scripts to query DisGeNET, OpenTargets and ClinVar.

OrphaNet identifiers (provided or inferred) are passed directly to DisGeNET to query disease-related genes and variants. A query to OpenTargets requires Experimental Factor Ontology (Malone et al., 2010) identifiers, which are obtained using the Ontology Lookup Service (Côté et al., 2010) API. Both DisGeNET and OpenTargets queries connect to the respective resources via their API and return a JSON file containing associated genes and their variants, including the association score provided by the respective platform. The workflow can specify the required level of the association score and the maximum number of genes to be returned (configurable parameters).

These lists are then combined with ClinVar data provided as a preprocessed file. The script goes through the obtained genes and variants and carries out pairwise comparison of ClinVar non-pathogenic variants (and thus also genes) with the DisGeNET and Open Targets genes and variants. The output is a combined list of genes and variants pertinent to given disease, together with a report showing the difference in representation of genes and variants across the resources. Additionally, the variants can be filtered by their allele frequency in several populations available in the Ensembl database through the Ensemble API endpoint (Cunningham et al., 2022) (configurable parameter).

### 2.2 Network of mechanisms

The disease-relevant lists of genes and variants are used to construct a network of mechanisms using three different resources: disease maps, pathways and text mining. To this end the list of gene variants is reduced only to the genes carrying their respective variants. The lists of genes and variant-derived genes are combined.

Disease maps offer standardized and diagrammatic descriptions of disease mechanisms (Mazein et al., 2018). They are independent resources developed by separate research groups, without a centralized architecture typically seen in pathway databases. To get systematic access to selected disease maps, we rely on MINERVA Net repository (Gawron et al., 2023), storing pointers to publicly accessible disease maps hosted using the MINERVA Platform (Gawron et al., 2016). Using MINERVA Net, we run Gene Set Enrichment Analysis for the disease-relevant gene list using the R package minervar (https://gitlab.lcsb.uni.lu/minerva/minervar) to identify areas of significance for a given gene list (Gawron et al., 2023). Results are constrained to a subset of the most enriched areas to avoid overpopulation (configurable parameter) and then exported together with their layout information, into CellDesigner SBML format (Kitano et al., 2005).

Pathway databases are another source allowing building detailed networks of disease-related mechanisms. Similarly to disease maps, they offer diagrammatic descriptions of mechanisms in molecular biology, but less relevant to a particular disease. We focused on two databases: WikiPathways and Reactome (Martens et al., 2021; Gillespie et al., 2022). These databases offer diagrams in GPML and SBML + layout + render formats, respectively, and the MINERVA Platform can convert them into a harmonized format (Hoksza et al., 2019a). We used the enrichR package, an R-based interface to the EnrichR server (Kuleshov et al., 2016) to calculate the enrichment in these databases. The exact selection of source databases (configurable parameter) is constrained to versions of WikiPathways and Reactome databases. Results are constrained to a subset of the most enriched areas to avoid overpopulation (configurable parameter) and then exported together with their layout information, into respective native formats. Importantly, for the GPML format, some details, e.g., custom images or interactions without reactant/product, are lost. For Reactome pathways, we have narrowed the results of enrichment to include only i) diagrams with layout and ii) the topmost diagrams, when nested diagrams are enriched.

Text mining allows to find relationships between genes which are not captured by expert curated resources, like disease maps or pathway databases. To fetch such potentially novel interactions between the disease-related genes, we used the STRING database (Szklarczyk et al., 2019) to request interactions of a pre-set minimal score (configurable parameter). STRING integrates primary and predicted interactions, includes annotated pathway knowledge, text-mining results and data obtained by ontology. The query to STRING retrieves the first *n* neighbors of the disease-relevant genes (configurable parameter). To improve the quality of text mining results, which are non-directional and may contain noise, we used contents of the OmniPath resource (Türei et al., 2016), which aggregates interactions from curated databases, including directionality and sign. The query to OmniPath retrieves all associations in the database for the disease-relevant genes, and only these interactions are kept that have both interaction partners in text mining results. Such directed network in a simple interaction format is then transformed into a diagram by applying the Fruchterman-Reingold algorithm in R package igraph (Csardi and Nepusz, 2006), and converted to GPML format using R package minervar.

Diagrams obtained in the steps above are then converted and merged using the MINERVA Platform API calls in the following order:

1. Disease map parts are merged into a single CellDesigner SBML diagram
2. Text mining diagram is converted from GPML to a CellDesigner SBML diagram
3. WikiPathways and Reactome diagrams are converted to CellDesigner SBML format and merged into a single diagram
4. Three components are finally merged into a single CellDesigner SBML diagram

This results in a diagram containing networks from the abovementioned sources, in a harmonized data and graphical format that is compatible with the MINERVA Platform.

### 2.3 Interactive prototype

We chose CellDesigner SBML format to harmonize and integrate above-mentioned resources. All source diagram formats (GPML, SBML + layout + render, simple interaction format) are translated using the MINERVA Platform conversion function (Hoksza et al., 2019a). Finally, single diagrams are combined using MINERVA API functionality for merging diagrams of the same type into a simple, mesh layout. The integrated map is bundled with the identified disease-related genes and variants to visualize them on top of the final diagram. The bundle is ready to be uploaded to the MINERVA Platform to host the generated disease map, making it interactive and allowing users to visually explore source data. For that, users will have to have access to an instance of the MINERVA Platform with curator or admin privileges. Alternatively, they can run MINERVA locally as a virtual machine (see https://minerva.uni.lu).

The workflow is implemented as a shell pipeline which can be configured by a parameters file where the user can set all the parameters mentioned above including the list of disease identifiers. The output of the pipeline is a ZIP file containing the disease map with genetic and variants overlays and can be imported into the MINERVA Platform. To demonstrate the maps discussed in Results, we use the pathwaylab.elixir-luxembourg.org instance of the MINERVA Platform hosted by the ELIXIR Luxembourg (https://elixir-luxembourg.org).

### 2.4 Gene expression analysis

For the retinitis pigmentosa example (Section 3.2), we calculated gene expression from PRPF31 retinitis pigmentosa (RP) patients iPSC-derived retinal organoids (Rodrigues et al., 2022). The data was downloaded from the Gene Expression Omnibus (GEO) database GSE206529 using the GEOquery R package (Davis and GEOquery, 2007). The data was filtered by t = D100, selecting only the samples related to RP patients carrying PRPF31 mutations (Cys247X) and controls (organoids derived from unaffected Cys247X family members). RNA-seq gene expression data were normalized with the Trimmed mean of M values (TMM) normalization method. Differentially expressed genes (DEG) were computed between Cys247X vs Control for cases having at least 5 counts per million (CPM) in at least one of the samples. All RNA-seq analysis was done using the edgeR package (Robinson et al., 2010). The DEGs were filtered for FDR-adjusted *p*-value <0.05 and absolute fold change >2, resulting in 371 entries. The dataset is available in the automap repository (https://gitlab.lcsb.uni.lu/minerva/automap/) under ‘associations/data/ORPHA791_DEGs’.

## 3 Results

### 3.1 Orphanet-based map of mechanisms in Kawasaki disease

**FIGURE 2:**
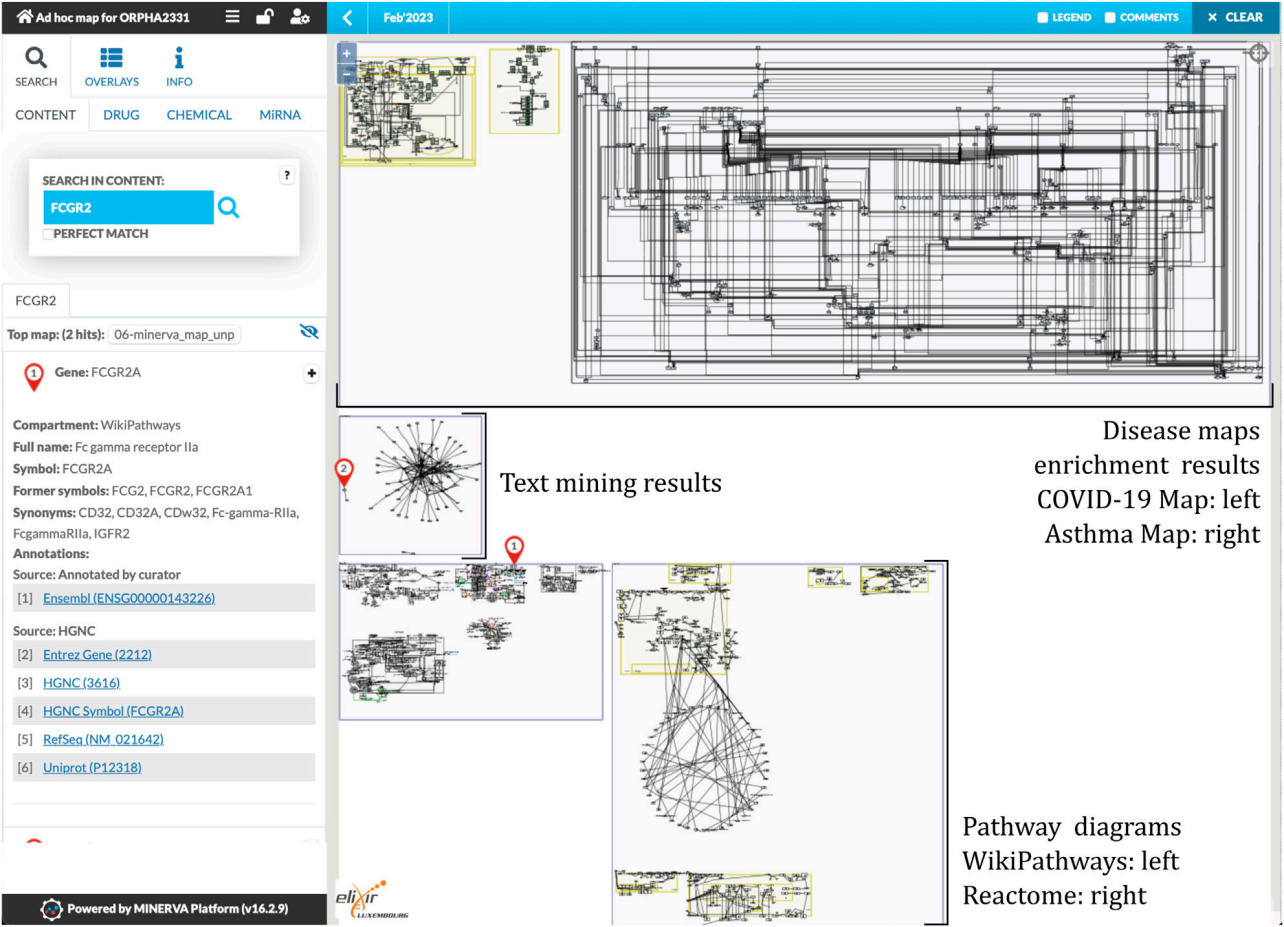
An overview of the ad-hoc map for the Kawasaki disease. The map includes three diagrams from two disease maps (two from the COVID-19 Disease Map (Ostaszewski et al., 2021), one from the Asthma Map (Mazein et al., 2021)), 192 interactions from text mining, and 5 pathways from both WikiPathways and Reactome databases. See https://pathwaylab.elixir-luxembourg.org/minerva/?id=adhoc_ORPHA2331.

In order to demonstrate an application of our workflow, we constructed an ad-hoc map of mechanisms for Kawasaki disease. To this end, we executed the workflow for the Orphanet identifier 2331 (https://www.orpha.net/ORDO/Orphanet_2331). The map is openly available at https://pathwaylab.elixir-luxembourg.org/minerva/?id=adhoc_ORPHA2331, hosted on a public instance of the MINERVA Platform. Figure 2 illustrates the overall layout of the map, and its main components. Below we discuss the results of particular steps of the workflow, leading to this outcome. Results of all the steps as well as the final build of the map are available in the Supplementary Materials.

#### 3.1.1 Assembly of components for the map

For the Kawasaki disease, and its Orphanet identifier, the pipeline has identified 56 disease-associated genes and three protein-coding variants based on the query to DisGeNET, OpenTargets and ClinVar. For the OpenTargets query, Orphanet identifier ORPHA2331 was mapped to EFO:0004246. Retrieved 56 genes were used to build the main components of the map. For the disease maps, we chose COVID-19 Disease Map (Ostaszewski et al., 2021) and the Asthma Map (Mazein et al., 2021) as maps having potentially related immune mechanisms (Lei et al., 2021; Makino et al., 2022). We would like to emphasize that the choice of these particular disease maps is for the demonstration purpose only and despite the cited article this relationship may be stretched. For the pathway enrichment, we chose the most recent collections of WikiPathways (WikiPathway_2021_Human) and Reactome (Reactome_2022) in the EnrichR server (Kuleshov et al., 2016). The workflow was configured to download up to three disease map diagrams, five pathway diagrams per selected database, and text mining interactions with at most 100 new neighbors (see parameters.sh in the Supplementary Materials).

The workflow retrieved three diagrams from the selected disease maps. One diagram from the COVID-19 Disease Map describes the coagulation pathway, two diagrams from the Asthma Map describe signaling in Th0 and epithelial cells, respectively. Text mining query resulted in a network of 192 interactions. Finally, after pathway enrichment, 5 diagrams from both WikiPathways and Reactome were retrieved. Figure 2 illustrates the result and the main components of the constructed ad-hoc map.

#### 3.1.2 Interactive map prototype with data overlays

**FIGURE 3:**
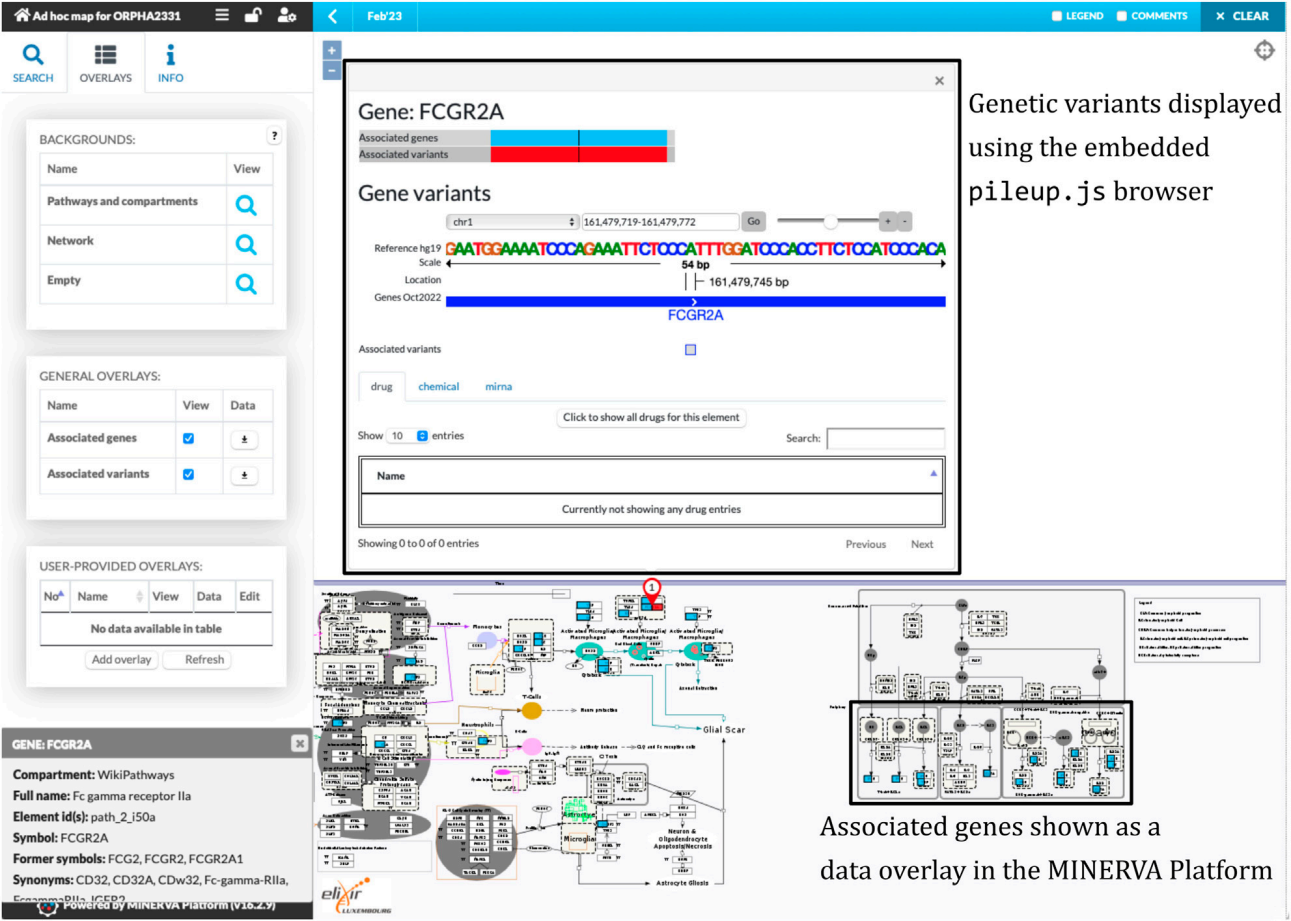
A view of the ad-hoc map with the visualized data overlays. One of disease-related variants in the FCGR2A gene is shown in the embedded pileup.js browser (Vanderkam et al., 2016) (top box). Disease-related genes, highlighted in blue, are shown as another visual overlay on top of the map (bottom box).

**FIGURE 4:**
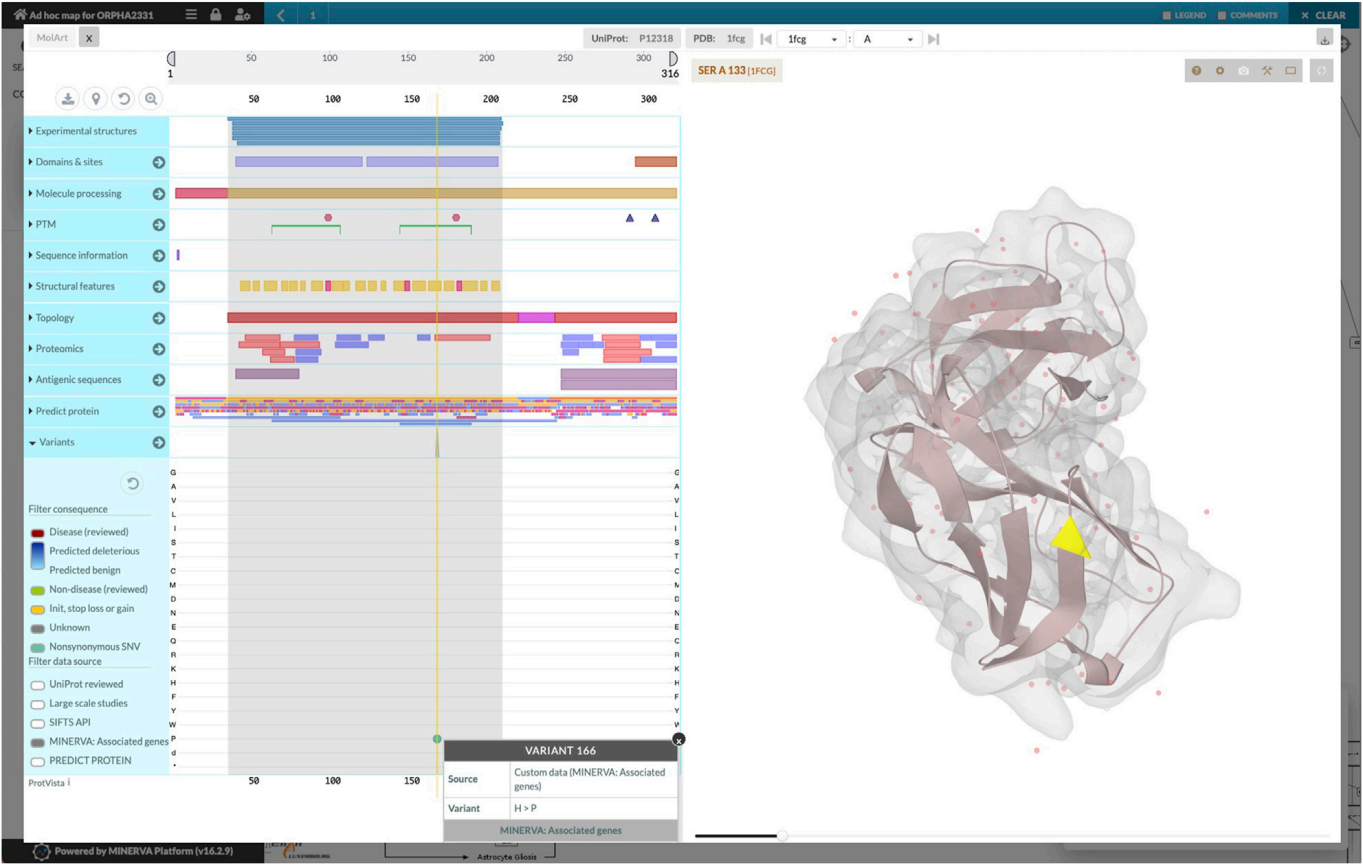
Protein structure view offered by MolArt visualization tool. MolArt (Hoksza et al., 2018) is integrated with the MINERVA Platform and available in the contextual menu for all proteins having UniProt annotation. Here, MolArt visualizes FCGR2A protein together with the position and context of the protein-coding mutation retrieved by the ad-hoc map building workflow.

The workflow combined the retrieved components into a single diagram, which was bundled together with disease-related genes and variants. We uploaded the bundle to the public instance of the MINERVA Platform, enabling easy browsing and search of this relatively large diagram. Moreover, the lists of genes and variants are available for visual exploration as data overlays. Figure 3 illustrates the details of such exploration. Map users can select the overlays in the left panel, highlighting relevant elements of the map. To visualize exact positions of genetic variants, the MINERVA Platform uses an embedded pileup.js browser (Vanderkam et al., 2016). Protein-coding variants are also visible in the protein structure visualization tool MolArt (Hoksza et al., 2018) integrated in the MINERVA Platform. The workflow annotates such variants, so they are visible together with other structural annotations, offering insight into functional consequences of the mutation. Figure 4 illustrates the protein structure view for the selected variant in FCGR2A gene. MolArt view is available in the map directly via the contextual menu for a given protein (right-click) making it straightforward to investigate protein function in the diagram.

#### 3.1.3 Interpretation of results

Kawasaki disease (KD), or mucocutaneous lymph node syndrome, is a disease of infants and young children, characterized by a multi-system inflammation with proinflammatory hypercytokinemia (Sakurai, 2019). Molecular pathophysiology is still unclear, however a number of implicated genes have been suggested in the passing years. Recent reviews point to enhanced T Cell activation (ITPKC, ORAI1, STIM1), dysregulated B Cell signaling (CD40, BLK, FCGR2A), decreased apoptosis (CASP3), and altered transforming growth factor beta (TGFB) signaling as major molecular mechanisms of KD (Bijnens et al., 2018; Kumrah et al., 2020). The map constructed with our workflow captures and highlights some of the pathways involved in KD. Mechanisms related to T Cell activation and TGFB signaling are captured by mechanisms imported from the asthma map (PLCB1-IP3 signaling) and WikiPathways describing different aspects of immune responses, including viral myocarditis, another interesting link to KD (Giryes and McGonagle, 2023). This is further reinforced by Reactome pathways covering a range of interleukin responses and pyroptosis. Next, mechanisms related to B Cell signaling are shown in text mining results (FCGR2A-BLK interaction). FCGR2A can also be found in a pathway describing immune response to spinal cord injury (WikiPathways, WP2431), but its role in this context would have to be further investigated. Finally, a diagram of coagulation-related mechanisms retrieved from the COVID-19 Disease Map proposes a link to the KD-like multisystem inflammatory syndrome following COVID-19 in children (Bukulmez, 2021). From this brief overview of recent literature, the ad-hoc map seems to be a reasonable starting point for developing a dedicated knowledge repository.

### 3.2 Orphanet-based map of mechanisms in retinitis pigmentosa

As a second application of our workflow, we constructed an ad-hoc map of mechanisms for retinitis pigmentosa (RP). The workflow was run for the OrphaNet identifier 791 (https://www.orpha.net/ORDO/Orphanet_791). The map is openly available at https://pathwaylab.elixir-luxembourg.org/minerva/?id=adhoc_ORPHA791, with a similar layout of components as in Figure 2. Below we discuss the results of the workflow. Detailed results of all the steps as well as the final build of the map are available in the Supplementary Materials.

#### 3.2.1 Assembly and setup of the map prototype

The pipeline has identified 64 disease-associated genes and 3004 protein-coding variants. Retrieved genes were combined with the RP-specific differentially expressed genes (see Section 2.4) and then used to build the main components of the map. For the disease maps, we chose Parkinson’s disease map (Fujita et al., 2014) and Aging map (https://progeria.uni.lu) to explore mechanisms of age-related degeneration, and COVID-19 map used earlier, following reports of potential involvement of COVID-19 in retinal pathology (Ichhpujani et al., 2022; D’Alessandro et al., 2022). We emphasize that the choice of these disease maps is for the demonstration purpose only and despite the cited articles this relationship may be stretched. Remaining setup was identical to the case above (see parameters.sh in the Supplementary Materials).

The workflow retrieved three diagrams from the selected disease maps. Two diagrams from the Parkinson’s disease map describe neuroinflammatory processes, and one diagram from the COVID-19 Disease Map describes the kynurenine metabolism pathway. Text mining query resulted in a network of 145 interactions. Following pathway enrichment, 5 diagrams from both WikiPathways and Reactome were retrieved.

Similarly to the ad-hoc map for Kawasaki disease, the workflow produced a map bundled together with disease-related genes and variants, which was then uploaded to the public instance of the MINERVA Platform. The list of DEGs was uploaded there as well, with log fold changes normalized to [-1,1] range for visual analysis of relative expression differences.

#### 3.2.2 Interpretation of results

**FIGURE 5:**
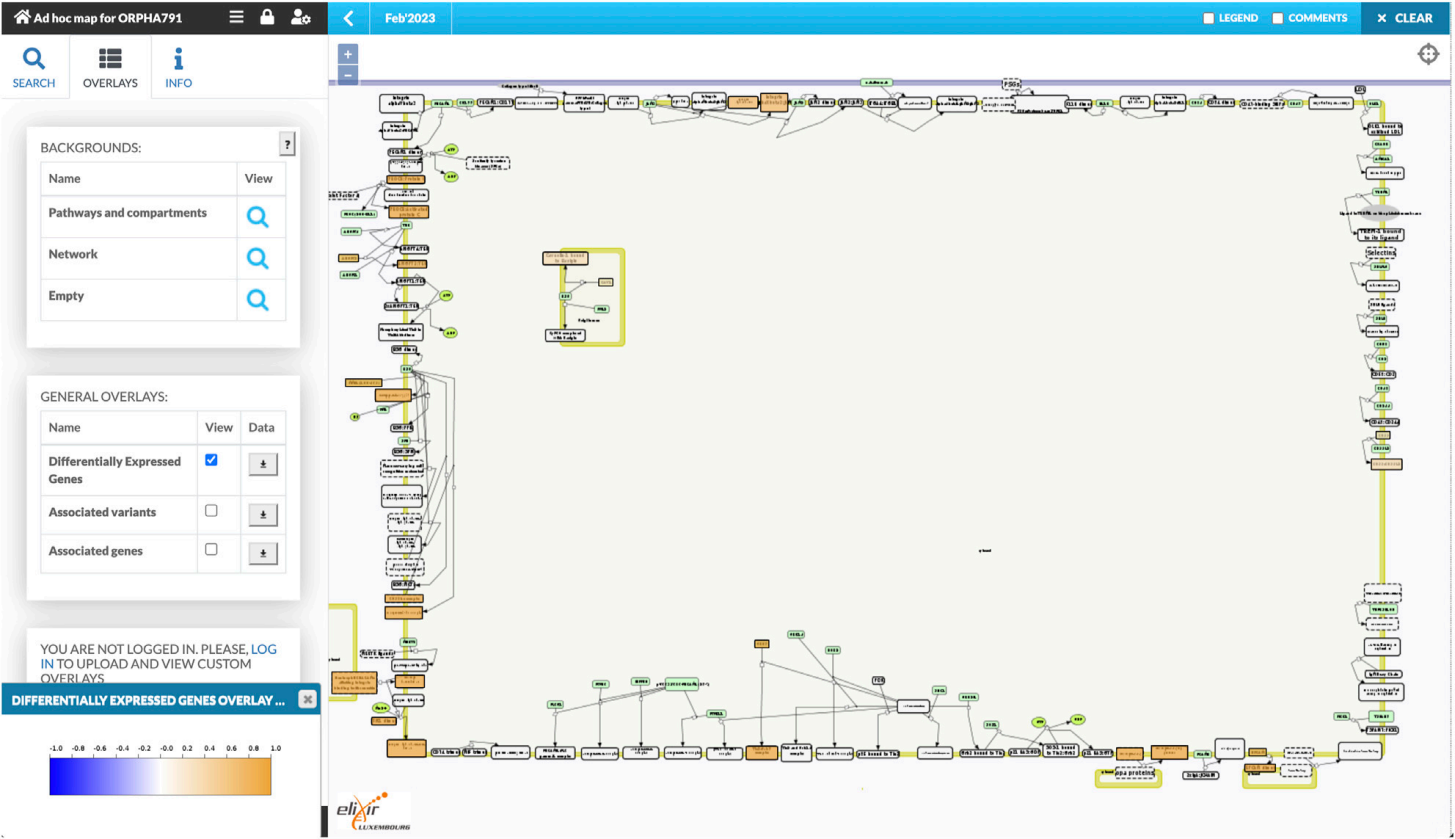
Visualization of differential gene expression in a fragment of the ad-hoc map. Multiple proteins and complexes of the “Cell surface interactions at the vascular wall” pathway (Reactome, R-HSA-202733) are differentially expressed in the RP dataset, indicating potentially dysregulated mechanisms.

Retinitis pigmentosa (RP) is a rare genetic disorder that causes the progressive degeneration of the retina photoreceptor cells (rod and cones). The heterogeneity of RP makes it not a single entity but rather a group of disorders, meaning that it can be caused by mutations in many different genes (Ayuso and Millan, 2010). Several cellular pathways have been implicated in the degeneration of photoreceptor cells in RP, including phototransduction, cell survival and metabolism, and vesicle trafficking (Ferrari et al., 2011). The phototransduction pathway is responsible for converting light into electrical signals, and mutations in genes such as RHO, RP1, and RDS that encode phototransduction proteins can lead to decreased visual sensitivity and progressive vision loss (Mannu, 2014). In the cell survival and metabolism pathway, which is responsible for maintaining the health of retinal cells including photoreceptor cells, mutations in genes like PRPF31 (Frontiers, 2021) and PRPH2 (Chakraborty et al., 2020) can cause increased cell death, abnormal disk formation, photoreceptor cell death, and retinal dysfunction. Protein trafficking within the photoreceptors is key to maintaining the overall retinal homeostasis. Mutations in genes encoding vesicle trafficking proteins can result in cellular dysfunction and increased cell death (Bales and Gross, 2016). The constructed RP Map from our workflow highlights the molecular mechanisms involved in RP pathophysiology. As we can see in the WikiPathways section on the middle-left, pathways describing processes involved in ciliopathies, with enrichment of gene variants associated with RP. The dysfunction of photoreceptors cilia proteins can result in various symptoms such as retinal degeneration and other pleiotropic phenotypes (Adams et al., 2007). Other key molecular mechanisms captured by the Reactome section, at the bottom-right side of the map, are Visual phototransduction, which is a well-defined hallmark process affected by RP (Ferrari et al., 2011), and Cell surface interactions at the vascular wall. The neural retina has a specific vascular network that delivers oxygen and nutrients to the neurons to keep them functioning properly. It has been described that metabolic issues in photoreceptor-neurons, which have the highest concentration of mitochondria in the body, can result in adaptive but ultimately harmful retinal vascular response (Fu et al., 2020). These fall in line with the high volume of differentially expressed genes (DEGs), from the GSE206529 dataset, observed in that section of the map depicted in Figure 5. Interestingly, the Visual phototransduction pathway is populated by variants and associated genes but not DEGS, which may be an indicator of protein malfunction but not altered expression in the genes encoding the proteins.

Neuroinflammation is an important player upstream of tissue degeneration in RP (Ortega and Jastrzebska, 2021); prominently represented by neuroinflammatory pathways from PD, emphasizing a potential link of PD-related alpha-synuclein to RP (D’Alessandro et al., 2022); kynurenine pathway upstream of prostaglandin synthesis links RP to COVID-19. Other pathways are missing from the map, including autophagy and apoptosis-related mechanisms (D’Alessandro et al., 2022). This may be caused by a limited scope of the diagram (only 3 disease map fragments and 5 pathways from each source); extending it may broaden the scope and refine the resource further; especially that the list of potential map elements and pathways includes these diagrams. Text mining results show an enrichment of DEGs associated with inflammatory processes, as well as potential regulators of CNGA1, a gene implicated in RP, and its associated mutations (Saito et al., 2021).

Taken all together, the ad-hoc RP map represents, as a big picture, a fair set of the processes implicated in the pathophysiology and development of RP. The workflow produced a general overview of relevant disease mechanisms, enabling the discovery of new connections and generation of knowledge.

## 4 Discussion

In this article we demonstrate a workflow for ad-hoc assembly of molecular interaction diagrams for rare diseases, enabling easy building of interactive, visual repositories supporting hypothesis building and data interpretation. Building such repositories requires resources which are often not available for particular rare disorders. To address this situation, we enable ad-hoc construction of maps of molecular mechanisms, based on limited prior information. Namely, an Orphanet identifier is required, which can be substituted by a list of HPO identifiers describing disease traits. To the best of our knowledge this is the first such approach to integrating different molecular interaction diagrams. By focusing on the visualization of large molecular diagrams, our work complements data-oriented workflows like ExpressVis (Liu et al., 2022a) or PaintOmics (Liu et al., 2022b), relying on pathway databases without combining their content. As demonstrated in two examples, the contents of constructed maps reflect state of the art molecular mechanisms of related diseases to the extent they can be used as starting points for further development.

The presented workflow automates a number of search and transformation steps, making it straightforward to run, and use its results. As the final diagram is generated automatically based on gene search, enrichment parameters and text mining, it is important to control the run parameters. In the examples discussed above, using a relatively constrained set of run parameters resulted in diagrams with 2512 unique elements and 2312 interactions (Kawasaki disease), and 2566 elements and 1304 interactions (retinitis pigmentosa). Broader searches can lead to an overgrown diagram which may be then difficult to use. This issue may be addressed by adjusting the scope of the created map by adding results of omics experiments relevant for a given disease, similarly to the resources cited earlier (Liu et al., 2022a; Liu et al., 2022b). Another approach may be referencing the recently released RDmap (Yang et al., 2021) to identify the proper parameters for the workflow.

Maps generated ad-hoc by our workflow can be further refined using available diagram editors, CellDesigner in particular. Such on-the-fly query for diagram components is similar to the functionality of Newt editor (Balci et al., 2021) querying of PathwayCommons resources (Rodchenkov et al., 2019). Our workflow preserves the layout of the original components, which may result in a patchwork of styles. We did not harmonize them for easier reference to the source diagrams, leaving the stylistic decisions to the end user. After eventual refinement, an ad-hoc map is meant to be visualized in the MINERVA Platform for visualization and analysis. MINERVA is a web-server having visual exploration capabilities, plugin architecture and extensive API, supporting network analysis and drug target search (Hoksza et al., 2019b). This way a generated map can be used as an input to advanced analytical workflows.

The work presented here has certain limitations. One of the challenges that remain to be solved is the scope of the constructed map. The choice of disease maps to query is based on a single Orphanet identifier, which may be too restrictive especially considering RD research. Although our workflow offers a possibility of identification of diseases based on their phenotypic traits, this is realized based on the HPO mapping in Orphanet. Addition of prior knowledge, like a custom list of genes or results of omics analysis, may be a possible improvement to this situation. Another challenge is harmonization of the outcome of the workflow, which currently is a combination of styles. The MINERVA Platform preserves the layout and rendering information from the source disease maps and pathway databases, but as these resources have different curators, styles of diagrams may vary significantly. Despite the final diagram being editable, refining it may be a challenging task. A possible approach here would be to split the resulting map into sub-diagrams based on their source and generate a top-level view with only disease-relevant genes involved. Finally, our workflow relies on external databases, whose contents may change. In effect the outcome may differ depending on when the workflow was executed.

## Data Availability

The datasets presented in this study can be found in online repositories. The names of the repository/repositories and accession number(s) can be found below: https://gitlab.lcsb.uni.lu/minerva/automap/, https://pathwaylab.elixir-luxembourg.org/minerva/export.xhtml?id=adhoc_ORPHA2331.
